# Changes in mosquito species and blood meal composition associated with adulticide applications

**DOI:** 10.1038/s41598-023-49494-3

**Published:** 2023-12-12

**Authors:** Dongmin Kim, Nathan D. Burkett-Cadena, Lawrence E. Reeves

**Affiliations:** Florida Medical Entomology Laboratory, University of Florida, Vero Beach, FL USA

**Keywords:** Ecology, Zoology, Diseases

## Abstract

Although adulticide application is a pillar in the integrated management of nuisance and vector mosquitoes, non-target effects of adulticide applications within ecosystems are a substantial concern. However, the impacts of adulticide applications on non-target organisms are not necessarily detrimental, and in some cases, may provide benefits to certain organisms or wildlife. Here, we hypothesized that adulticide applications have beneficial non-target impacts on vertebrate wildlife through reduced biting pressure. To test this, we collected mosquitoes from ultra-low volume Permanone-treated (intervention) and untreated (reference) areas and assessed mosquito abundance and diversity, and abundance of blood-engorged female mosquitoes. We performed DNA barcoding analysis on mosquito blood meals to identify host species. Our results demonstrated a significant reduction in mosquito abundance by 58.9% in the intervention areas, taking into account the reduction in reference areas. Consequently, this decline led to a 64.5% reduction in the abundance of blood-engorged females. We also found a temporal dynamic of mosquito composition driven by mosquito control actions in which different mosquito species became dominant at treated sites while composition at reference areas remained similar during the same period. The present study suggests that the beneficial effects of mosquito control treatments for humans extend to other vertebrates, which represents an unstudied and rarely recognized non-target impact.

## Introduction

Mosquitoes are the primary vectors for numerous arboviruses causing a wide spectrum of diseases in humans that range from mild symptoms to death^1,2^. State and local government agencies play an important role in protecting public health by monitoring viral transmission and engaging in mosquito control activities on a year-round basis. In the U.S., government agencies tasked with reducing the impacts of vector and nuisance mosquitoes apply an integrated approach to mosquito management. One of the elements of this approach used to suppress mosquito population levels and limit the potential of arbovirus transmission is the application of adulticides (i.e., pesticides that target adult mosquitoes). Adulticide application has several advantages including availability of adult mosquito-targeting pesticides and rapidity of treating large areas with precise concentrations and minimum volumes of chemicals. However, there are disadvantages, including the potential for unintended impacts on non-target organisms.

Non-target impacts of mosquito adulticides in nature are poorly characterized. Much of the research on this topic focused on laboratory-based sensitivity of non-target insects to commonly used adulticide active ingredients or their commercial formulations. However, such laboratory-based susceptibility experiments (e.g., bottle bioassays), usually involving a limited range of model organisms (e.g., European honeybee, butterflies), may not reflect the real-world non-target impacts of adulticide applications in complex and diverse natural systems^3^. The potential ecological impacts of mosquito adulticide applications may differ with respect to biological/behavioral traits, ecological interactions, environments, and control regimes^4^. For example, a previous study identified that the circadian activity patterns and resting behaviors (e.g., nocturnal roosting behavior among vegetation, in forest canopies, or underground) may minimize the exposure-related effects of adulticides on diurnal non-target organisms^5^. Further, field studies have failed to find clear and consistent evidence of a significant decline in butterfly species diversity and abundance in areas subjected to repeated adulticide treatments, compared to untreated areas^6,7^. The impacts of adulticide applications on non-target organisms may not always be detrimental, and in some cases, may benefit organisms in certain circumstances. Recent studies provided important clues suggesting that mosquito adulticide treatment inferred an indirect benefit to a butterfly species (*Danaus plexippus*) by reducing parasitoid pressure on eggs on host plants where adulticide had been applied^7^. Therefore, the actual impacts of mosquito adulticide applications on organisms are likely to be nuanced and complex, consisting of varied direct and indirect, and beneficial and detrimental effects.

Mosquito-borne pathogens can be a significant challenge to wildlife health either directly or indirectly. Several mosquito-vectored viruses including Rift Valley fever, eastern equine encephalitis, and Japanese encephalitis are transmitted among wildlife populations by species of the genera *Aedes*, *Culex,* and *Anopheles*^8^. For example, the Rift Valley fever virus adversely affects various wildlife including ruminants, monkeys, and rodents, causing high mortality in newborns and abortions^9^. Japanese encephalitis virus-infected boars experience testicular edema and more than 50% reproductive loss^2^. Florida scrub jays, an imperiled and federally listed species, are exposed to avian pox virus (Poxviridae), causing die-offs^10^. West Nile virus (WNV) infections in birds lead to various outcomes depending on the taxon, ranging from high mortality in crows, other corvids, and raptors, to sublethal or benign effects in many songbirds and doves^11–13^. Avian malaria (*Plasmodium relictum*, among other species) transmitted by *Culex quinquefasciatus* has caused significant devastation to birds that have led to population decline and even extirpations and extinctions^14^. Also, these arbovirus-infected wildlife (e.g., birds) are considered amplifying hosts of several arboviruses responsible for the long-distance transport and sustained local transmission^15^.

Harassment caused by nuisance mosquitoes is often underestimated, despite several studies emphasizing its significance to humans and animals. Skin reactions to mosquito bites often come with general symptoms including itching and inflammation, although the severity can vary from person to person. Scratching the bite site can lead to hyperpigmentation and superinfection in some individuals, which can significantly impact their quality of life^16^. Specifically, intense mosquito harassment can have dramatic consequences on outdoor activities like hiking, fishing, and hunting, leading to restricted time for tourism^17^. These disruptions can have a notable impact on the economy in the U.S., as tourism plays a vital role in various regions and communities.

The adverse effects of mosquito harassment extend beyond humans. Mosquito harassment can exert a significant impact on the movements, reproduction, quality of habitats, and survival of birds and non-human mammals^18–22^. For example, during July and August, reindeer exhibit a tendency to migrate to higher elevations on mountains to avoid insect harassment, even when those areas are subject to lower food quality and high anthropogenic disturbances^23^. Nest-bound hatchling birds and brooding females are particularly susceptible to mosquito feeding which has been linked to nest failure in the critically endangered waved albatross^24^. Among livestock, mosquito bites can irritate nursing sows, leading to reduced livestock fitness, weight loss, higher rates of overlays, and an increase in piglet deaths^25^. Therefore, the application of mosquito-targeting adulticides to the environment is expected to confer benefits to humans and likely to other mosquito host animals in the form of reduced harassment and the related reduction in transmission of wildlife pathogens via decreased biting rate.

The potential benefit of reduced mosquito feeding from wildlife in areas treated with mosquito-targeting adulticides has not been assessed in nature, but studies suggest that this may be an unintentional consequence of adulticide applications. For example, on a smaller scale, the treatment of barn owl nest boxes with mosquito adulticides was associated with reduced numbers of blood-engorged mosquitoes and higher survivorship among fledglings^22^. Several federally or state-listed imperiled vertebrate species including the Florida scrub jay, snail kite, grasshopper sparrow, and whooping crane, may be more sensitive to the effects of mosquito feeding and mosquito-vectored pathogens due to small population sizes. If these impacts are reduced by mosquito control measures, it could be considered a beneficial non-target impact of mosquito control adulticide application. Here, we hypothesized that mosquito adulticide applications have the potential to confer benefits to wildlife populations through reduced mosquito feeding which is a neglected and potentially positive ecological impact of mosquito control actions. To test this, we collected mosquitoes before and after adulticide application at treated and untreated field sites to assess change in mosquito abundance, diversity, and abundance of blood-engorged female mosquitoes. Through DNA barcoding-based blood meal analysis, we inferred the biting rates from each vertebrate host species detected.

## Results

**Figure 1:**
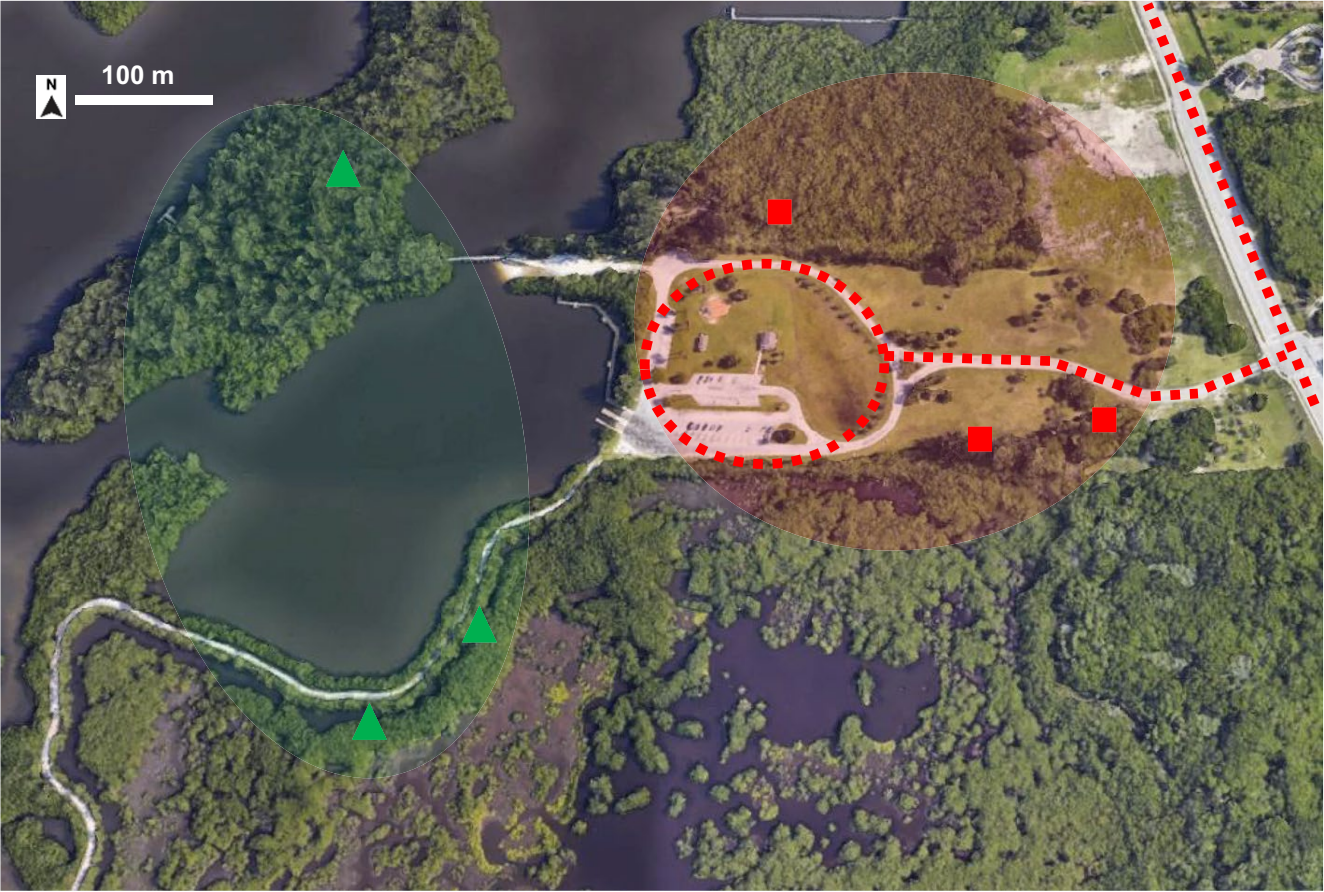
Example of placement of collection sites at Round Island Park (RIP), Indian River County, Florida. Sampling sites are located relative to an Indian River Mosquito Control District spray route (red dotted lines), with intervention areas (red squares) adjacent to the spray route, and reference areas (green triangle) located in areas expected to be unaffected by adulticide applications. The satellite image extracted from Google Earth (Version 7.3.2.5776)-Own work.

Mosquito sampling was conducted using CDC light traps baited with 1.5 kg dry ice before and after truck-mounted ULV applications (Permanone 30–30) at five distinct sites within Indian River County, Florida, in June and September 2021. These sites represented both suburban and rural areas, and within each site, multiple trapping locations were included (Table 1). The intervention areas were located in close proximity to the designated spray routes, while the reference areas were carefully selected to remain unaffected by the direct impacts of the adulticide spray (Fig. 1).

**Table 1:** Summary of mosquito collection sites and coordinates in Indian River County, Florida, 2021. For each trial, three or four sampling replicates (day) were performed in succession.

Collection site	Habitat type	Sampling dates (range)	Latitude, longitude	Trials (*n*)	Sampling locations (*n*)
South county park (SCP)	Suburban	Aug. 23 –26, 2021	27° 35′ 30.2″ N, 80° 24′ 24.3″ W	1	6
Indian river fairground (IRF)	Rural	Jun. 29–Jul. 2, 2021	27°44′24.1″N, 80° 26′ 44.4″ W	3	6
		Aug. 2–6, 2021			
		Aug. 10–14, 2021			
South fire station (SFS)	Suburban	Jul. 20–23, 2021	27° 35′ 16.3″ N, 80° 27′ 19.2″ W	1	6
Oslo riverfront conservation Area (ORCA)	Rural	Jun. 15–19, 2021	27° 35′ 12.8″ N, 80° 22′ 09.6″ W	3	6–10
		Jul. 12–16, 2021			
		Aug. 16–21, 2021			
Round island park (RIP)	Rural	Aug. 31–Sep. 3, 2021	27° 33′ 41.8″ N, 80° 19′ 39.9″ W

**Figure 2:**
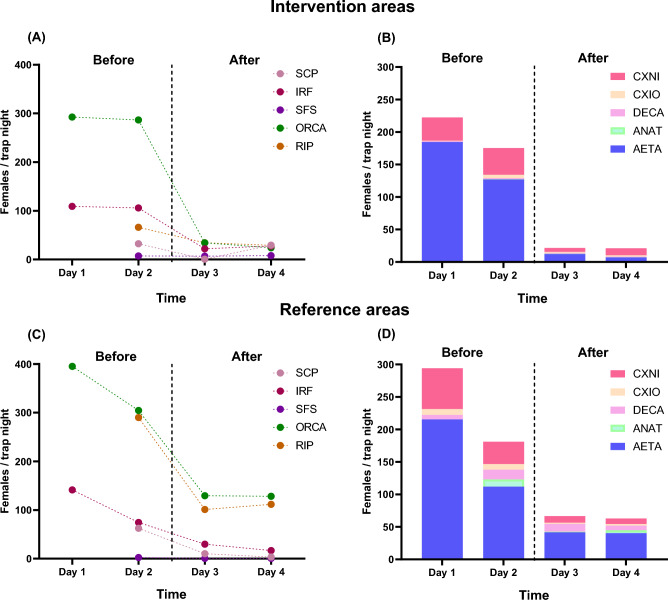
The effect of adulticide applications on the abundance of mosquitoes collected in Indian River County, Florida. Adulticide applications were conducted in the intervention areas (**A**) and (**B**), while no adulticide applications were performed in the reference areas (**C**) and (**D**). “Before” indicates mosquito collections (colored points) made during the one or two days preceding adulticide applications. The dashed line indicates the point of time at which the adulticides were applied at the intervention areas. “After” indicates mosquito collection during the two days immediately following adulticide applications. Abbreviations in (**A**) and (**C**): SCP, IRF, SFS, ORCA, and RIP represent South County Park: one trial of three replicates (n = 3), Indian River Fairground: three trials of four replicates (n = 12), South Fire Station: one trial of three replicates (n = 3), Oslo Riverfront Conservation Area: three trials of four replicates (n = 12), and Round Island Park: one trial of four replicates (n = 4), respectively, in Indian River County, Florida. ﻿Species abbreviations in (B) and (D): CXNI *Culex nigripalpus*, CXIO *Culex iolambdis*, DECA *Deinocerites cancer*, ANAT *Anopheles Atropos*, AETA *Aedes taeniorhynchus*.

Across the five sites, we collected a total of 25,682 female mosquitoes consisting of eight genera and 23 species. The effects of adulticide application on mosquito abundance and diversity were relatively variable from site to site (Table 2). Overall, the total number of mosquitoes was significantly reduced by 84.6% (*p* < 0.0001) in the intervention area and 64.9% (*p* = 0.0001) in the reference area following the adulticide application. The adjusted estimated percent reduction in intervention areas, accounting for the reduction in reference areas, is 58.9%. The abundance of *Aedes taeniorhynchus*, the species targeted by these applications in intervention areas, experienced a significant decrease of 91.8% (estimated percent reduction: 76.7%). Among all trap locations at each site (n = 5), overall mosquito numbers at the Indian River Fairgrounds site (IRF) (*p* = 0.0022) and Oslo Riverfront Conservation Area site (ORCA) (*p* = 0.0146) were significantly different between the intervention and reference areas before and after adulticides application (Fig. 2A). There was a significant difference pre-and post-treatment between the number of mosquitoes collected at the reference (untreated) areas of South County Park (SCP) (*p* = 0.201), IRF (*p* = 0.0022), and ORCA (*p* = 0.0428) (Fig. 2C). More than 91.2% of all collected mosquitoes were species of the genera *Aedes* and *Culex*, with *Ae. taeniorhynchus* being the most abundant species across all sites. The highest mosquito diversity was found in ORCA (22 species), while the lowest diversity was found in the South Fire Station (SFS) (10 species). The IRF and ORCA sites, representing rural areas, were dominated by species of the genera *Aedes* and *Culex***.** Collections in SFS, representing a suburban area, were also dominated by *Aedes* and *Culex* species*,* while collections in SCP, another suburban area, were dominated by *Culex* and *Psorophora***.** Traps in Round Island Park (RIP), also in a rural area, were primarily dominated by *Deinocerites cancer*, and to a lesser extent, species of *Anopheles*. Across all intervention areas, 82.6% (19/23) and 78.3% (18/23) of the total number of mosquito species were identified before and after the application, respectively. In the reference area, 95.7% (22/23) and 82.6% (19/23) of the species were collected before and after the application (Table 2), respectively.

**Table 2:** Total number of mosquitoes before and after adulticide application, collected by CDC Miniature light trap baited with dry ice at five sites in Indian River County, Florida, 2021.

Mosquito species	Intervention areas^a^					Reference areas^b^					Total
	Before		After		% Change	Before		After		% Change	
	Adulticide		Adulticide			Adulticide		Adulticide			
	Total (N)	Mean^c^	Total (N)	Mean		Total (N)	Mean	Total (N)	Mean		
*Aedes aegypti*	5	0.1	12	0.2	140.0	4	0.1	2	0.0	− 50.0	23
*Aedes albopictus*	12	0.3	30	0.5	150.0	37	0.8	29	0.5	− 21.6	108
*Aedes infirmatus*	9	0.2	2	0.0	− 77.8	20	0.4	8	0.1	− 60.0	39
*Aedes pertinax*	0	0.0	0	0.0	–	1	0.0	2	0.0	100.0	3
*Aedes sollicitans*	7	0.2	2	0.0	− 71.4	30	0.6	0	0.0	− 100.0	39
Aedes taeniorhynchus*	6820	148.3	558	9.6	− 91.8	7249	147.9	2381	41.1	− 67.2	17,008
*Anopheles atropos*	17	0.4	18	0.3	5.9	355	7.2	154	2.7	− 56.6	544
*Anopheles crucians complex*	46	1.0	33	0.6	− 28.3	110	2.2	47	0.8	− 57.3	236
*Anopheles quadrimaculatus s.l*	4	0.1	4	0.1	0.0	1	0.0	4	0.1	300.0	13
*Culex cornator*	108	2.3	48	0.8	− 55.6	65	1.3	37	0.6	− 43.1	258
*Culex declarator*	1	0.0	0	0.0	− 100.0	4	0.1	2	0.0	− 50.0	7
*Culex erraticus*	21	0.5	15	0.3	− 28.6	28	0.6	10	0.2	− 64.3	74
*Culex iolambdis*	158	3.4	103	1.8	− 34.8	426	8.7	123	2.1	− 71.1	810
*Culex nigripalpus*	1806	39.3	498	8.6	− 72.4	2175	44.4	549	9.5	− 74.8	5028
*Culex quinquefasciatus*	6	0.1	5	0.1	− 16.7	0	0.0	1	0.0	–	12
*Deinocerites cancer*	71	1.5	63	1.1	− 11.3	596	12.2	553	9.5	− 7.2	1283
*Mansonia titillans*	18	0.4	1	0.0	− 94.4	9	0.2	4	0.1	− 55.6	32
*Psorophora ciliata*	0	0.0	0	0.0	–	2	0.0	0	0.0	− 100.0	2
*Psorophora columbiae*	41	0.9	15	0.3	− 63.4	57	1.2	20	0.3	− 64.9	133
*Psorophora ferox*	2	0.0	1	0.0	− 50.0	6	0.1	0	0.0	− 100.0	9
*Uranotaenia lowii*	1	0.0	1	0.0	0.0	7	0.1	3	0.1	− 57.1	12
*Uranotaenia sapphirina*	0	0.0	0	0.0	–	2	0.0	0	0.0	− 100.0	2
*Wyeomyia mitchellii*	0	0.0	0	0.0	–	4	0.1	3	0.1	− 25.0	7
Total	9153	199.0	1409	24.3	− 84.6	11188	228.3	3932	67.8	− 64.9	25682
No. species	19		18			22		19			23

*Targeted species.

^a^Intervention area: Adulticide applications were performed.

^b^Reference area: No adulticide applications were performed.

^c^Mean number of adult female mosquitoes collected per trap-night.

**Figure 3:**
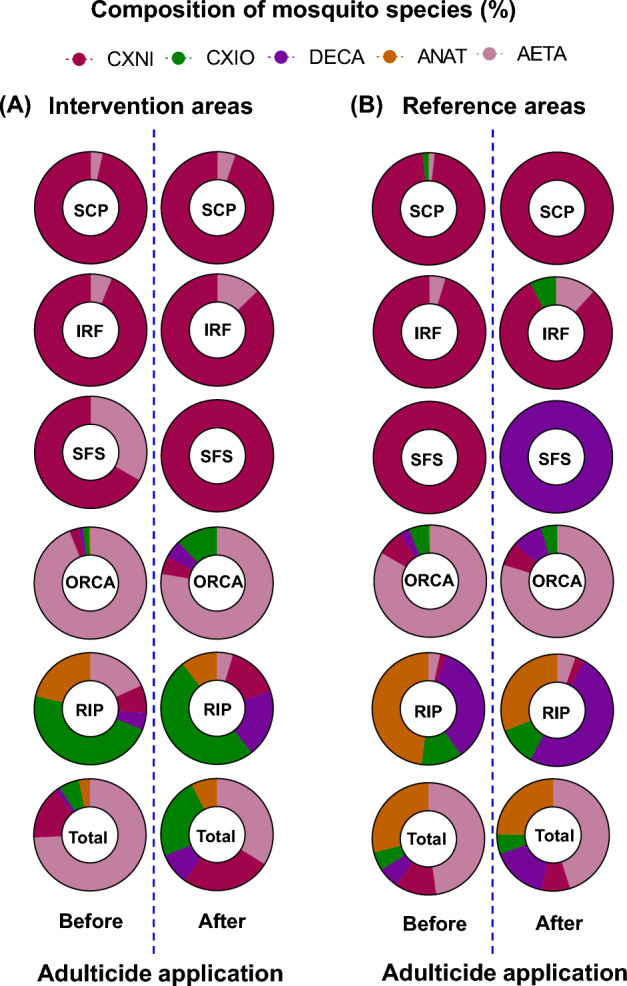
The effect of adulticide applications on the composition of the most common mosquito species collected at five rural and suburban sites in Indian River County, Florida. SCP, IRF, SFS, ORCA, and RIP represent South County Park, Indian River Fairground, South Fire Station, Oslo Riverfront Conservation Area, and Round Island Park, respectively. Adulticide applications were conducted only in the intervention areas. Mosquito composition before and after adulticide applications are separated by vertical dashed lines. Species abbreviations: AETA *Aedes taeniorhynchus*, CXNI *Culex nigripalpus﻿*, DECA *Deinocerites cancer*, CXIO *Culex iolambdis*, ANAT *Anopheles atropos*.

Overall, there was no significant reduction in mosquito diversity at intervention and reference areas following adulticide application. The most common species across all sites were *Ae. taeniorhynchus*, *Anopheles atropos*, *Culex iolambdis*, *Culex nigripalpus*, and *Deinocerites cancer*, and all were observed across all five sites. Differences in species proportions were observed after adulticide application in the combined intervention areas (Fig. 3A). Both a significant decline (X^2^ = 19.1, *p* < 0.0176, 54.3%) in the proportional abundance of *Ae. taeniorhynchus* and an increase (X^2^ = 5.6, *p* < 0.0001, 69.3%) in proportional abundance of *Cx. nigripalpus* were found following adulticide applications. The proportion of mosquito species at reference areas remained similar and overall consistent during the same period (Fig. 3B), but the dominant species at the SFS site changed from *Cx. nigripalpus* to *De. cancer* after the application.

Blood-engorged females were sampled using a combination of CDC light traps, modified large-diameter aspirators, and pop-up resting shelters. All collected blood-engorged mosquitoes were identified to the species level, and each mosquito's blood meal was preserved and assessed for the extent of digestion. Host identifications were determined using PCR-based blood meal analysis.

A total of 151 blood-engorged mosquitoes were sampled using CDC light traps (n = 111), aspirators (n = 39), and resting shelters (n = 1). Species of *Culex* (*Cx. quinquefasciatus*, *Cx. nigripalpus*, *Cx. iolambdis*, and *Cx. erraticus*), *Aedes* (*Ae. albopictus* and *Ae. taeniorhynchus*), and *Psorophora* (*Ps. columbiae*) were found in the combined areas. *Aedes taeniorhynchus* (n = 72), *Cx. nigripalpus* (n = 34) and *Cx. iolambdis* (n = 21) constituted 84.1% of the total blood-engorged mosquito sample throughout the study period. Specimens with the extent of digestion estimated to be BF1 (blood meal estimated to have been obtained within 24 h of collection) constituted 39.7% of the total blood fed mosquito sample, while 24.50% and 35.76% were BF2 (24–48 h) and BF3 (48–72 h), respectively. A total of 42 (27.8%) blood-engorged mosquitoes were collected at the intervention (treated) areas, while 109 (72.2%) were collected at reference (nontreated control) areas. Across all intervention areas, 31 (73.8%) blood-engorged mosquitoes were collected prior to the application, and 11 (26.2%) were collected after. Across all reference areas, 81 (74.3%) blood-engorged mosquitoes were collected prior to the adulticide application, while 28 (25.7%) were collected after. The total number of collected blood-engorged mosquitoes was significantly reduced by 64.5% in intervention areas and 65.4% in reference areas after the application.

Overall, 70/151 (46.4%) blood meals were identified at the species level (Table 3). Fourteen blood meals were identified from intervention areas, and of these, only three blood meals (25.0%) were collected following an adulticide application, while eleven were collected prior to treatment. Comparatively, 56 blood meals were identified from mosquitoes collected in reference areas, and of these, 20 (35.7%) were collected after the application. In total, 23 host species were identified from blood meals (Table 3) representing amphibians (one species), birds (ten species), mammals (ten species), and reptiles (two species). Specifically, blood meal analysis showed that collected mosquitoes fed either from birds (45.5%) or mammals (45.5%) before the application, but only mammals (100.0%) were detected after the application at the intervention areas. The reference area showed a wider range of blood meal sources, compared to the intervention area. Mosquitoes fed on birds (17.9%), mammals (79.5%), and reptiles (2.6%) before the application, but blood meal sources were detected from amphibians (5.8%), birds (17.9%), mammals (79.5%), and reptiles (2.6%) after the application at reference areas. *Aedes taeniorhynchus* and *Ps. columbiae* fed predominantly on mammals (82.4% and 100.0%, respectively) but *Cx. nigripalpus* fed on a wide range of hosts including mammals (59.1%), birds (27.3%), and reptiles (13.6%). *Culex iolambdis* fed mainly upon birds (88.9%) and a single amphibian-derived blood meal was detected.

**Table 3:** Summary of host species (n = 23) identified from mosquito blood meals (n = 70) by DNA barcoding before and after adulticide applications in intervention areas and reference areas at five sites in Indian River County, Florida, 2021.

Host class	Host species	Mosquito species											Mosquito species											Total
		CXNI*			CXIO			AETA			PSCO		CXNI			CXIO			AETA			PSCO		
		Intervention areas^a^											Reference areas^b^											
		B^c^	A	—	B	A	—	B	A	—	B	A	B	A	—	B	A	—	B	A	—	B	A	
Amphibian	*Rana sphenocephala*	–	–		–	–		–	–		–	–	–	–		–	1		–	–		–	–	1
Bird	*Ardea herodias*	–	–		1	–		–	–		–	–	–	–		–	–		–	–		–	–	1
	*Bubo virginianus*	1	–		–	–		–	–		–	–	–	–		–	–		–	–		–	–	1
	*Butorides virescens*	–	–		2	–		–	–		–	–	–	–		1	–		–	–		–	–	3
	*Cairina moschata*	–	–		–	–		–	–		–	–	–	–		–	–		–	1		–	–	1
	*Gallus gallus*	–	–		–	–		–	–		–	–	1	–		–	–		–	–		–	–	1
	*Grus canadensis*	–	–		–	–		–	–		–	–	–	–		–	–		–	1		–	–	1
	*Megascops asio*	1	–		–	–		–	–		–	–	–	2		–	–		2	–		–	–	5
	*Nyctanassa violacea*	–	–		–	–		–	–		–	–	–	–		2	2		–	–		–	–	4
	*Nycticorax nycticorax*	–	–		–	–		–	–		–	–	–	–		–	–		1	–		–	–	1
	*Zenaida macroura*	–	–		–	–		–	–		–	–	–	1		–	–		–	–		–	–	1
Mammal	*Bos taurus*	–	–		–	–		–	–		–	–	2	–		–	–		–	–		–	–	2
	*Dasypus novemcinctus*	–	–		–	–		–	1		–	–	1	–		–	–		3	2		–	3	10
	*Didelphis virginiana*	–	–		–	–		–	–		–	–	2	–		–	–		2	1		–	–	5
	*Felis catus*	–	–		–	–		1	–		–	–	1	–		–	–		2	–		–	–	4
	*Glaucomys volans*	–	–		–	–		–	–		–	–	1	–		–	–		–	–		–	–	1
	*Homo sapiens*	–	–		–	–		1	–		–	–	–	–		–	–		5	–		–	–	6
	*Neotoma floridana*	–	1		–	–		–	–		–	–	1	–		–	–		–	–		–	–	2
	*Procyon lotor*	–	–		–	–		1	–		–	–	1	–		–	–		–	–		–	–	2
	*Sylvilagus floridanus*	–	–		–	–		2	1		–	–	1	–		–	–		3	3		1	1	12
	*Sylvilagus palustris*	–	–		–	–		–	–		–	–	2	–		–	–		–	–		–	–	2
Reptile	*Anolis sagrei*	1	–		–	–		–	–		–	–	1	–		–	–		–	–		–	–	2
	*Coluber constrictor*	–	–		–	–		–	–		–	–	–	1		–	–		–	1		–	–	2
	Total	3	1		3	0		5	2		0	0	14	4		3	3		18	9		1	4	70

*Species abbreviations: CXNI *Culex nigripalpus*, CXIO *Culex iolambdis*, AETA *Aedes taeniorhynchus*, PSCO *Psorophora columbiae*.

^a^Intervention area: Adulticide applications were performed.

^b^Reference area: No adulticide applications were performed.

^c^B: Before adulticide applications; A: After adulticide applications.

## Discussion

We measured mosquito abundance at five sites before and after mosquito adulticide treatments (Table 1). Each site had intervention areas directly impacted by the adulticide application and reference areas not directly impacted. We found a significant reduction in mosquito abundance at both intervention (84.6%) and reference (64.9%) areas following adulticide application (Table 2), which remained relatively stable for at least two days post ULV spray (Fig. 2). When adjusting for the reduction in reference areas, the estimated percent reduction in intervention areas is 58.9%. Similarly, previously published studies found temporary declines (29.2–90.0%) in mosquito abundance at intervention areas after mosquito adulticide treatment^26–28^. Relatively high degrees of resistance to pyrethroid active ingredients are widely observed in Florida^29–31^. This underscores the significant point that the type/formulation of the adulticide and the peak activity of certain mosquito species during specific timings of adulticide sprays directly impact their susceptibility level. Decreases in mosquito abundance at reference (ULV untreated sites) were unexpected, but not without precedent. If mosquito adulticides drifted beyond the targeted area, mosquito dispersal away from untreated reference areas could account for the reductions in mosquito abundance we found at reference sites following treatments. It is also conceivable that the single ULV spray conducted on a specific night was less effective, and both the intervention and reference sites experienced natural population declines that were unrelated to the ULV spray. We selected multiple trapping locations within adulticide intervention and reference areas, representing suburban and rural landscapes, to ensure random sampling. The reference areas were located at substantial distances (~ 150–700 m) from the ULV spray path. Our preliminary assessment identified that the effectiveness of adulticides started to diminish approximately 50–75 m from the spray path in open-field settings. A previous study also indicated that adulticide deposition and concentration (Permanone) significantly decreased over 50 m from the adulticide spray path^32^. While the reasons for the significant reduction in mosquito density at reference areas were unclear, factors such as natural mosquito movements, dispersal, and local meteorological conditions (e.g., ambient temperature, relative humidity, atmospheric turbulence intensity) may influence population dynamics and occurrence. Mosquitoes can disperse over substantial distances within these relatively limited areas, which means adulticide treatments in one area may indirectly impact surrounding areas by reducing the total number of mosquitoes that could move to untreated areas. This dynamic population-level response to ULV treatment is well explained by the robust rebound and cascading effects^26,33^. Additionally, wind velocities influencing particle distribution and impingement may lead to increased adulticide drift, causing droplets to move farther from the application site than expected^34^.

We found a temporary change in mosquito composition at intervention areas driven by adulticide applications that can result in shifts in species dominance. Most notably, the adjusted estimated reduction in the composition of *Ae. taeniorhynchus* mosquitoes in the intervention areas, factoring in the reduction in reference areas, amounts to 51.6% among the five most prevalent mosquito species. Meanwhile, mosquito composition at reference areas pre- and post-adulticide application remained similar (Fig. 3). Major changes in dominant species in a given area are crucial to shaping the community, which could potentially affect arbovirus transmission and control measure planning^35,36^. *Aedes taeniorhynchus* comprised 74.3% of the total mosquitoes collected in combined intervention areas before adulticide application. This information is valuable for interpreting our results related to benefits to wildlife because *Ae. taeniorhynchus* are aggressive biters and known to serve as potential bridge vectors of viral encephalitides^37,38^ and *Hepatozoon* parasites for wildlife (e.g., reptiles, amphibians)^39^. *Aedes taeniorhynchus* feeds primarily from mammals^40^ but will feed on other vertebrate orders as well, including large reptiles. Large numbers of this mosquito have been observed feeding from American crocodiles in the Florida Everglades and from Galapagos tortoises on the Galapagos Islands^41,42^. Reduced *Ae. taeniorhynchus* abundance may relieve biting pressure on mammalian and other wildlife hosts. Declines in *Ae. taeniorhynchus* were accompanied by an increased total proportion of *Cx. nigripalpus*. Previous literature described this species as an opportunistic feeder^43^ that feeds from birds, mammals, and reptiles^44,45^, frequently feeding on birds in tree canopies^46^. The effect of height on adulticide-induced mortality in mosquitoes has not received much attention and the vertical stratification of *Cx. nigripalpus* may facilitate the survival of a subset of susceptible individuals if adulticides do not reach individuals located in canopies. A study found that the highest survival (100%) was observed for caged mosquitoes placed at the greatest vertical height above the adulticide treatment area (i.e., 7 m) and smallest distance from the ULV spray path (25 m), with significantly higher survival than both 1 and 4 m heights^5^. It is possible that increased wind speed at high heights above ground level decreases the effectiveness of adulticides, or that ULV sprays do not drift vertically to such heights, both potentially resulting in an increased mosquito survival rate following ULV application. It is not surprising that an increased proportion of *De. cancer* was found following the ULV application. Adult *De. cancer* are known to occupy a unique microhabitat, inside land crab burrows, providing lower environmental pressures (e.g., temperature, predation)^47^, which may produce a temporal refuge reducing adulticide exposure if the timing of adulticide applications is asynchronous with their circadian above-ground activity period. Circadian activity patterns may also play a role in explaining some of this variation. For example, mosquito species for which activity peaks are before or after adulticide applications may avoid the most severe detrimental impacts.

We quantified the abundance of blood-engorged females using multiple trapping methods including CDC miniature light traps, large-diameter aspirators, and pop-up resting shelters between ULV-treated and -untreated sites to estimate the change in the frequency of biting rate. The present study found that the total number (n = 151) of collected blood-engorged mosquitoes was reduced by 64.5%, which corresponds to the reduction in population density by 58.9% (the estimated percent reduction) in the intervention areas. This suggests that mosquito adulticide treatments confer benefits to wildlife similar to those conferred to humans. Arbovirus transmission is a complex interplay between the virus, host, and vectors, and these relationships are further shaped by multiple biotic and abiotic factors^48^. Vector abundance, biting contact, and survivorship are fundamental quantifiable parameters, expected to be in a positive-linear relationship with arbovirus transmission frequency^49^. Robust field evidence suggested that ULV applications suppressing vector populations led to the immediate decline of transmission incidence in the following four weeks^50^, while a rebound effect on the population was observed afterward. We found that more than 87.4% of total blood-engorged mosquitoes belonged to the genera *Culex* and *Aedes*, with *Ae. taeniorhynchus* (47.7%) and *Cx. nigripalpus* (22.5%) being the most abundant species across sites. Particularly, blood-engorged *Cx. nigripalpus*, the primary enzootic and epizootic vector of several arboviruses, was significantly reduced by 90.9% and 70.6% in intervention and reference areas following adulticide application, respectively (estimated percent reduction: 69.1%). The capture rate of blood-engorged females can be influenced by various factors including age, trapping methods, and the availability (density) of suitable hosts^51^. For example, Jones et al.^52^ revealed that mosquito susceptibility to pesticide application rises with age. Specifically, the younger age group of mosquitoes showed a higher resistance to the pesticide, which could potentially result in an increase in their blood feeding frequency. The number of blood-engorged mosquitoes captured in our study may not be correlated to the probability of their natural transmission success. However, the importance of blood meal acquisition to pathogen transmission in natural settings cannot be ignored.

Approximately half (46.4%) of blood meals collected during the study period were able to be identified at the species level. This identification success rate is relatively low compared to other studies^41,44^ using similar methods and may have been caused by the advanced digestion extent of the majority (~ 60% of collected blood meals scored as BF2 and BF3) of collected blood meals. Relatively low sensitivity of host identification was correlated with increased blood meal scores (BF2 and 3), which constituted 85.2% of unidentified blood meals. This correlation is likely due to the fact that as the blood meal is digested over time, the host DNA that is the target of blood meal analysis becomes increasingly degraded until it is undetectable after ~ 48 h post-feeding^53,54^. The sample of identified hosts consisted of 23 species including amphibians, birds, mammals, and reptiles (Table 3). Among those that were identified, several species were detected that are phylogenetically similar to imperiled species or are themselves protected species (e.g., Florida woodrat, sandhill crane, and several heron species). Several birds identified in this study are highly susceptible to infection with arboviruses. For example, the majority of blood meals derived from birds were identified as heron species that are known as amplifying hosts for Japanese encephalitis serocomplex including St. Louis encephalitis virus (SLEV)^55^. Previous studies indicated that emerging WNV and avian pox virus appear to be capable of causing fatal diseases in the great horned owl (*Bubo virginianus,* n = 1) and eastern screech owl (*Megascops asio,* n = 5), both species that play an important role in the ecosystem by regulating small mammal (e.g., rodents) populations^56,57^. Eastern cottontail (*Sylvilagus floridanus*) was the most utilized mammalian host overall in this study (n = 12) and is susceptible to Myxoma virus^58^ vectored by mosquitoes. Interestingly, the total number of host species identified was significantly reduced by 66.7% in intervention areas following adulticide applications. This result supports the idea that not only was there a reduction in mosquito population size and diversity at intervention areas but also in mosquito host-seeking and feeding behavior, which may translate to reduced biting pressure on wildlife. Mosquito-host interactions could have been interrupted by adulticide impacts on the olfactory system in cases of mosquito sublethal exposure to ULV adulticide^26^ which are commonly found in various other arthropod taxa including European honeybees^59^. Patterns of mosquito host association within an ecological community can affect respective roles between host and vector in pathogen transmission and lead to the understanding of interactive roles within unique ecological settings (i.e., adulticide application).

As the vectors of pathogens, mosquitoes can transmit viruses and other pathogens among wildlife, in some cases, leading to high mortality or other effects among populations. As blood-feeding insects, their bites can reduce the success of nestling birds, cause birds to abandon nests, or impact the movements or survival of mammals. We hypothesized that ULV adulticide may confer benefits to wildlife in the form of reduced mosquito harassment and blood-feeding relevant to the transmission of mosquito-vectored pathogens. Our findings indicate that mosquito control treatments had an immediate and significant impact on mosquito communities in terms of abundance and species composition. Specifically, the population of *Ae. taeniorhynchus*, the species targeted by these ULV applications in intervention areas, experienced a substantial decline of 76.7% in estimated percent reduction. As a result, nuisance caused by mosquitoes and their blood-feeding activities decreased, providing benefits to humans and potentially extending to other vertebrate animals. A limitation of our work is that we were unable to determine the effect of adulticide application on the abundance of gravid mosquitoes which may have a different response or susceptibility to ULV adulticide exposure, contributing to a potential population rebound effect. A more in-depth analysis of the effects of mosquito adulticide application on mosquito populations at different vertical and horizontal distances needs to be investigated. Our study does not provide the adulticide resistance status of mosquito populations in intervention areas where adulticides have been extensively deployed over long periods of time, and resistance may be expected to develop. This may explain the increased total proportion of some mosquito species (e.g., *Cx. nigripalpus*) following ULV application. Regardless, these results suggest that adulticide treatments do not impact all mosquito species uniformly and may promote the increased abundance of some species over others that are more susceptible to treatments. Fully characterizing the potential benefits conferred to vertebrate wildlife will improve understanding of the realistic impacts of adulticide applications on ecosystems and will help with effective mosquito management to both protect human health and mitigate threats to wildlife against mosquito harassment and vector-borne disease.

## Methods

### Study sites and mosquito sampling

Mosquito sampling was performed before and after routine adulticide treatments by Indian River Mosquito Control District (IRMCD). These treatments were scheduled in response to a high prevalence of mosquitoes, particularly species like *Ae. taeniorhynchus*, or as part of regular measures during the summer mosquito season. Mosquitoes were collected at five sites in Indian River County, Florida: South County Park (SCP), Indian River Fairground (IRF), South Fire Station (SFS), Oslo Riverfront Conservation Area (ORCA), and Round Island Park (RIP) (Table 1). These locations represented suburban and rural areas of Indian River County. Sampling took place in June and September 2021. Each field site was identified in coordination with the IRMCD using established GIS-based spray routes to inform the selection of suitable areas that were treated by adulticide. Selected areas were considered adulticide intervention zones, located immediately adjacent to spray routes, not more than 50 m from the route (Fig. 1). Nontreated control sites were selected in close proximity (150–700 m) to the spray routes as reference areas expected to be unimpacted by the adulticide spray. Mosquito sampling was performed on one or two days (depending on the prior notice IRMCD personnel had of when and where treatment would occur) before and for two days after each treatment. The timing of treatments varied across sites, and only one site was treated/sampled on a given date. The IRMCD treated the spray route nearest the collection sites using an ultra-low volume Permanone 30–30 (30% permethrin, Bayer Environmental Science, NC, U.S.) truck-mounted spray, applied at a rate of 0.36 oz per acre.

Several methods were used to sample mosquitoes. Mosquito abundance and diversity were measured by CDC light traps baited with 1.5 kg dry ice. Equal numbers (three to five) of traps were placed in intervention or reference areas. The traps were each operated for three hours per night (11:30 p.m.–2:30 a.m.), regulated by automatic timers (BioQuip Products Inc., CA, U.S.). The collected adult mosquitoes were freeze-killed by placing them into a cooler with dry ice and then stored at − 20 °C. Blood-engorged females were separated and identified into species using morphological characters and were stored in a − 80 °C freezer. On the morning of each CDC trap retrieval, mosquitoes were sampled with modified large-diameter aspirators^60^ and pop-up resting shelters^61^ intended to target blood-engorged females. On each trapping night, six to ten pop-up resting shelters were set at each collection site on the evening prior to the trap retrieval. Resting mosquitoes were removed from the shelters and collected into a collection cup manually by compressing the entire resting shelter. The resting shelter collection cups were transported back to the laboratory in a cooler with dry ice and stored in a − 80 °C freezer until processing. Each aspiration lasted for five minutes, and during this time, the running aspirator was moved through or over potential resting sites (e.g., tree buttresses, rock crevices, animal burrows, and undergrowth) to collect mosquitoes flushed from their resting sites. Following each aspiration, mesh bags containing collected mosquitoes were labeled with the site and date and were transported in a cooler from the field to the laboratory.

### Mosquito identification and molecular identification of blood meals

All collected mosquitoes were identified using keys to the morphological identification of Florida mosquitoes^62^. Mosquito specimens that could not unambiguously be identified to species using morphological keys because of damage to diagnostic characters (e.g., scales, wing patterns) were identified by DNA barcoding following the protocol described in Reeves et al.^63^ Host identifications were determined using PCR-based blood meal analysis. After identification, each mosquito blood meal was preserved on Whatman Flinders Technology Associates (FTA) blood cards, and the extent of digestion was estimated as described by Reeves et al.^53^. Blood meal DNA from the FTA Cards was extracted using the Hot Sodium Hydroxide and Tris (HotSHOT) method^53^. Mosquito host DNA templates were amplified in a polymerase chain reaction (PCR) with primers designed to amplify the DNA barcoding region of the vertebrate cytochrome *c* oxidase subunit I (COI) gene^41^. Each reaction was performed in a 20 µl final volume consisting of 10 µl 2.0X Apex Taq RED Master Mix, 0.75 µl forward primer Mod_RepCOI_F (10 µM), 0.75 µl reverse primer VertCOI_7216_R (10 µM), 7.5 µl ultra-pure water, and 1 µl extracted DNA. Each reaction was run under the following conditions: 95 °C for 3 min, followed by 40 cycles of 95 °C for 40 s, 45 °C for 30 s, and 72 °C for 3 min, with a final extension step of 72 °C for 7 min. PCR products were visualized on a 1.5% agarose gel to confirm positive reactions and sent to Eurofins Genomics (Louisville, KY, U.S.) for DNA sequencing using the chain-termination method^64^. The resulting DNA sequence files were edited for quality in the bioinformatic software Geneious Prime Version 11.0.6 (Biomatters, Inc., CA, U.S.). To determine host species, each sequence was submitted to the Barcode of Life Data Systems (BOLD) identification tool (http://www.boldsystems.org). Blood meal sequences with > 98% similarity to a reference sequence were considered conspecific. Identified blood meals were used to infer the host composition of mosquito blood meals by site and between treated and nontreated control areas.

### Statistical analysis

**Figure 4:**
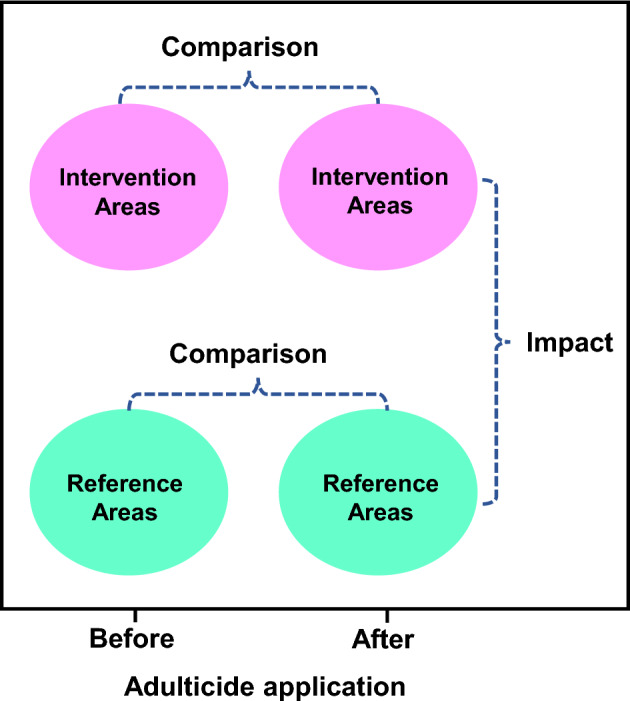
The schematic depicts the BACI (Before-After, Control-Impact) framework, which involves the evaluation of both intervention and reference areas before and after the implementation of pesticide measures. The impact of the measures was determined by comparing the two after situations and calculating the difference obtained. The image has been adapted from the study by Carpenter et al.^67^.

Estimated percent reduction was calculated using Mulla's formula^65^ as follows: % reduction = 100 − (R_1_/I_1_ × I_2_/R_2_) × 100, where R_1_ and R_2_ are the mean number of mosquitoes collected in reference areas before and after adulticide applications, respectively, and I_1_ and I_2_ are the mean number of mosquitoes collected in intervention areas before and after adulticide applications, respectively. The BACI (Before-After, Control-Impact) method (Fig. 4), used for assessing the effects of environmental and ecological interventions^66^, was employed in this study. A Wilcoxon signed-rank test was conducted in JMP Statistics, Version 15.0 (SAS Institute Inc., Cary, NC, USA) to detect changes in mosquito abundance before and after the pesticide application in the intervention areas. These measurements were then compared with the before and after data of the same variables at reference areas. By evaluating the differences between the intervention and reference areas, the impact generated by the interventions was determined. For the effect of adulticide on mosquito compositions among the most common species, data were normalized to the same magnitude from 0 to 1 and evaluated using separate chi-square randomization tests. For all analyses, results were considered significant if *p* ≤ 0.05.

## Data Availability

The datasets utilized and/or analyzed during the present study are accessible from the corresponding author upon reasonable request.
